# The Role of Group and Save Testing Prior to Emergency Laparoscopic Appendicectomy

**DOI:** 10.7759/cureus.74898

**Published:** 2024-12-01

**Authors:** Vinita Chaudhari, Hossain Mohammad, Umair Khan

**Affiliations:** 1 General Surgery, United Lincolnshire Hospitals Trust, Lincoln, GBR

**Keywords:** bleeding risks, blood group and save, cost-effectiveness analysis, emergency laparoscopic appendicectomy, general and laparoscopic surgery, laparoscopic appendicectomy, nhs, preoperative evaluation, preoperative group and save

## Abstract

The routine use of preoperative group and save (G&S) blood tests in emergency laparoscopic appendicectomies has been a standard yet often unquestioned practice. However, with the advancements in laparoscopic techniques and the low risk of intraoperative bleeding, is this precaution necessary? Analysing 276 emergency appendicectomy cases over a year, our study revealed that no transfusions were required due to surgical complications. Nevertheless, routine G&S testing causes considerable financial and resource strains, consuming valuable time and delaying treatment. These results question the value of this practice and suggest a need for reevaluation. Can we achieve better resource management while maintaining patient safety? By proposing targeted testing for high-risk individuals, this article sparks debate on optimising preoperative strategies.

## Introduction

The group and save (G&S) blood test is used to determine an individual’s blood type (ABO and Rhesus group) and to preserve a sample for potential transfusion. Its primary purpose before surgeries such as laparoscopic appendicectomies (LAs) is to ensure blood availability in the event of significant haemorrhage. However, during LAs, bleeding can occur from the appendicular artery within the mesoappendix, which can usually be controlled laparoscopically. Advancements in laparoscopic instrumentation, techniques, and training have made basic laparoscopic operations safer, with minimal blood loss during surgery [1-5]. Currently, the United Kingdom lacks national guidance on routine preoperative G&S testing for LAs; National Institute for Health and Care Excellence guidelines for elective surgery exclude routine G&S testing, emphasising clinical judgment by surgical and anaesthetic teams [6]. We aim to determine whether G&S testing is conducted as a precautionary measure rather than a necessity by auditing all appendicectomies (including diagnostic laparotomies and cases converted to open appendicectomies) performed over a 12-month period and by assessing whether observed rates of significant blood loss align with published findings.

## Materials and methods

This is a retrospective analysis of patients undergoing emergency appendicectomies at Lincoln County Hospital within the United Lincolnshire Hospitals Trust (ULHT). Data were obtained through theatre records (Theatreman software, CompuTix Systems, Inc., Centennial, CO, USA.) of all laparoscopic procedures performed from 1 February 2023 to 29 February 2024 inclusive. Any laparoscopic procedures not related to the removal or the assessment of the appendix were excluded. The sample comprised 276 cases, which, along with LAs, also included laparoscopic procedures converted to open and diagnostic laparoscopies for suspected appendicitis. All age groups were included in this study.

Once the sample had been identified, data on all individuals’ G&S statuses, preoperative haemoglobin levels, and the need for blood transfusions were obtained via WebV software (Northern Lincolnshire & Goole NHS Foundation Trust, South Yorkshire, UK). Theatre notes were reviewed for each case to determine whether any significant bleeding occurred during the operation. In the event that an individual required a transfusion, data were cross-referenced across hospital records, theatre notes, and discharge summaries to verify the indication and context of any transfusion. The rates of transfusions, conversions to open procedures, and intraoperative bleeds amongst the cohort were calculated (as percentages).

We consulted the blood transfusion practitioner within the Blood and Pathlinks Pan Trust department locally, who provided us with information and data regarding the policies, usage, and costs of blood products (RBCs) and the G&S test. This enabled us to perform a financial analysis by calculating the cumulative cost of G&S tests and blood transfusions over the study period.

## Results

**Figure 1:**
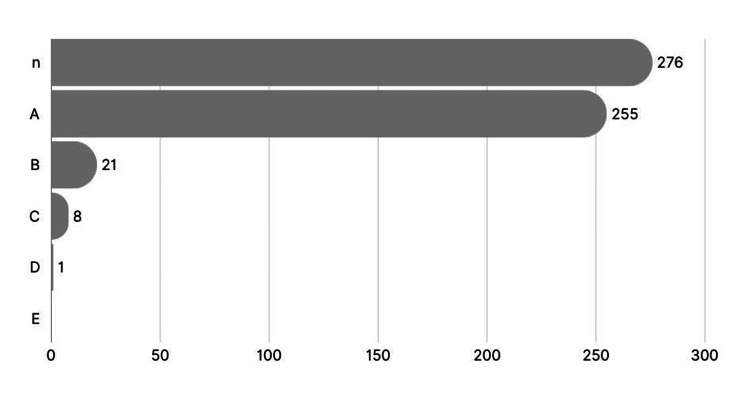
Key findings from 276 emergency appendicectomies: insights into testing and transfusion needs n: total number of patients (276), A: number of patients who underwent G&S testing (255), B: number of patients who did not undergo G&S testing (21), C: number of procedures converted to open (8), D: number of patients who received postoperative blood transfusions for non-surgical causes (1), E: number of patients who received blood transfusions due to operative complications (0) G&S: group and save

The study analysed 276 emergency appendicectomy cases and revealed no instances of operative bleeding necessitating blood transfusion. Of the total cases, only one patient (0.36%), who had pre-existing chronic anaemia and myelodysplastic syndrome, required a postoperative blood transfusion due to their underlying condition rather than surgical complications. Conversion to open procedures occurred in eight cases (3.63%), yet none of these patients required transfusions. Additionally, 21 patients (8%) underwent surgery without G&S testing, though the rationale for omitting this step remains unclear. Figure 1 provides a bar chart illustrating the distribution of key results, providing a clear visualisation of the data trends.

Table 1 presents a detailed breakdown of the costs for various blood tests and transfusion components at ULHT (data from 2021). The total cost for conducting group, antibody screen, and crossmatch is £26.78 per person, amounting to approximately £6,838.90 for 255 patients. This analysis provides a clear overview of cost allocation across the tests, which can aid in budgeting and resource planning. While additional factors such as inflation or consumable costs (e.g. vacutainers) have not been considered here, they could further add to the overall expenses. Furthermore, the cost of standard red cells (including universal donor O negative) within our trust is £183.78 per unit.

**Table 1:** Cost analysis of blood testing and transfusion components

Test	Cost (£)
Group	11.64
Antibody screen	10.48
Crossmatch	4.66
Standard red cells (per unit)	183.78

## Discussion

The findings of our study demonstrate a negligible rate of transfusion, which highlights the low risk of significant bleeding associated with LAs. Serious intraoperative complications are rare (with major vessel injury historically reported at around 0.07-0.12%), and this is almost always associated with the use of a Veress needle or closed trocar insertion for induction of pneumoperitoneum. With an open approach to establishing pneumoperitoneum, this risk drops to almost zero. Bleeding may also arise from direct injury to anterior abdominal wall vessels such as the inferior epigastric [1,7-10]. This is typically minor and manageable; however, it can become severe, especially if the bleeding remains undetected [11,12].

Additionally, given the nature of the aforementioned studies, which involve data from multiple surgeons across different hospitals, the low rates of bleeding observed can be attributed to the combined efforts of different surgical teams, each operating with their own techniques. This includes their preferred methods of establishing pneumoperitoneum; a study by Catarci et al. including 12,919 cases showed that the small risk of bleeding (0.18%) associated with the use of a Veress needle in a closed approach to pneumoperitoneum can be reduced to (0.09%) by employing an open approach [8]. This suggests that minor adjustments in the surgical approach can further mitigate the risk of vascular injury.

Furthermore, a study shows transfusion risk remains low (<1%) across age groups, with a slight increase in older patients. Transfusion risk is primarily associated with preexisting conditions, such as anticoagulation treatment or anaemia, rather than the procedure itself. Therefore, type and screen indications should be guided by individual clinical characteristics rather than age [13].

Our findings align with most of the published literature on the topic. For instance, a systematic review by Fadel et al. analysed five studies with a combined sample size of 2,952 patients undergoing appendicectomies [14]. Despite the larger sample size compared to our study and the inclusion of data from multiple hospitals, only four patients (0.1%) required a perioperative blood transfusion. This finding highlights the consistency of outcomes across different studies and suggests that bleeding risk is not significantly influenced by variations in sample size or the settings in which the studies are conducted. Furthermore, multi-centre studies naturally demonstrate that the rate of bleeding is consistent regardless of the skill and experience of the surgeons performing the procedures, further validating the repeatability of these findings.

A recent retrospective analysis conducted by Al-Musawi et al. examined data from LAs and laparoscopic hernia repairs over a four-year period. Of the 1,891 patients included in the study, 1,462 underwent LA procedures [15]. The analysis revealed that £47,298 was spent to ensure G&S testing was performed prior to these surgeries, yet only one patient (0.053%) required a transfusion.

Farrell et al. also discussed the financial implications of carrying out G&S testing prior to LAs. About 645 emergency appendicectomies over a three-year period were reviewed, of which 42 (6.5%) were converted to open procedures. Despite the larger sample size and higher conversion rate compared to our study, only one patient (0.2%) required a transfusion. Farrell et al. estimated that using a targeted approach to crossmatching rather than routine G&S testing would have saved £22,345 in their department alone. When extrapolated across the whole NHS, this could have amounted to savings of £1.1 million. Moreover, the study highlights the significant time and staff resources used by unnecessary G&S testing, emphasising the burden on healthcare systems without gaining proportional clinical benefits [16].

We propose that G&S testing should be reserved for high-risk patients. For the remaining patients, educating theatre staff on the major haemorrhage protocol and using O-negative (universal donor) blood (at £183.78 per unit in our trust) could provide a more cost-effective approach should an unexpected bleed occur.

This study has several limitations. Firstly, we could not determine why 21 patients (8%) in our cohort had not undergone G&S testing, although this is likely due to human error on the ward. Secondly, while our findings support reserving G&S testing for high-risk patients, we were unable to establish clear, evidence-based criteria for identifying these individuals, highlighting the need for further exploration in this area. Thirdly, our single-centre study over 12 months has a relatively small sample size, limiting its validity compared to larger multicentre studies. Additionally, we did not assess the long-term outcomes of omitting G&S testing, nor did we account for indirect costs such as staff time and patient flow.

## Conclusions

This study emphasises the exceptionally low risk of significant bleeding during LAs, even in cases converted to open procedures. Tailoring G&S testing to high-risk groups, supported by robust haemorrhage protocols and the use of universal donor blood, offers a pragmatic and cost-effective alternative. These findings call for the development of evidence-based guidelines to eliminate redundant practices, promoting efficiency and sustainability in surgical care.

## References

[1] Magowan D, Smith L, Williams G (2020). Utility of preoperative ‘group and save’ samples in laparoscopic appendicectomy. Bull R Coll Surg Engl.

[2] Ramasamy S, Bylapudi SK, Kumar S (2024). A retrospective study of routine preoperative blood grouping and saving in laparoscopic surgeries: a minimally utilized expenditure. Cureus.

[3] Kaafarani HM, D'Achille J, Graham RA (2011). Non-trocar related major retroperitoneal bleeding during laparoscopic appendectomy. World J Emerg Surg.

[4] Thomson PM, Ross J, Mukherjee S, Mohammadi B (2016). Are routine blood group and save samples needed for laparoscopic day case surgery?. World J Surg.

[5] Kleif J, Thygesen LC, Gögenur I (2021). Moving from an era of open appendectomy to an era of laparoscopic appendectomy: a nationwide cohort study of adult patients undergoing surgery for appendicitis. Scand J Surg.

[6] (2016). Routine Preoperative Tests for Elective Surgery. https://www.nice.org.uk/guidance/ng45.

[7] Barrett-Lee J, Vatish J, Vazirian-Zadeh M, Waterland P (2018). Routine blood group and antibody screening prior to emergency laparoscopy. Ann R Coll Surg Engl.

[8] Catarci M, Carlini M, Gentileschi P, Santoro E (2001). Major and minor injuries during the creation of pneumoperitoneum. A multicenter study on 12,919 cases. Surg Endosc.

[9] Sandadi S, Johannigman JA, Wong VL, Blebea J, Altose MD, Hurd WW (2010). Recognition and management of major vessel injury during laparoscopy. J Minim Invasive Gynecol.

[10] Fruhwirth J, Koch G, Mischinger HJ, Werkgartner G, Tesch NP (1997). Vascular complications in minimally invasive surgery. J Pelvic Surg.

[11] Bakshi GK, Agrawal S, Shetty SV (2000). A giant parietal wall hematoma: unusual complication of laparoscopic appendectomy. JSLS.

[12] Fernández EM, Malagón AM, Arteaga I, Díaz H, Carrillo A (2005). Conservative treatment of a huge abdominal wall hematoma after laparoscopic appendectomy. J Laparoendosc Adv Surg Tech A.

[13] Ghirardo SF, Mohan I, Gomensoro A, Chorost MI (2010). Routine preoperative typing and screening: a safeguard or a misuse of resources. JSLS.

[14] Fadel MG, Patel I, O'Leary L, Behar N, Brewer J (2022). Requirement of preoperative blood typing for cholecystectomy and appendectomy: a systematic review. Langenbecks Arch Surg.

[15] Al-Musawi J, Reece I, Chen JY (2023). Perioperative group and save testing are not routinely indicated for emergency laparoscopic appendicectomy and laparoscopic hernia repairs: a North West London retrospective study. J Perioper Pract.

[16] Farrell IS, Hall J, Hill J (2020). Cost analysis of blood group and antibody screening for emergency appendicectomy: should we stop?. World J Lap Surg.

